# Operator-Based Triboelectric Nanogenerator Power Management and Output Voltage Control

**DOI:** 10.3390/mi15091114

**Published:** 2024-08-31

**Authors:** Chengyao Liu, Ryusei Shimane, Mingcong Deng

**Affiliations:** Department of Electrical and Electronic Engineering, Tokyo University of Agriculture and Technology, 2-24-16 Nakacho, Koganei-shi 184-8588, Tokyo, Japan; s240969y@st.go.tuat.ac.jp (C.L.); s249039x@st.go.tuat.ac.jp (R.S.)

**Keywords:** triboelectric nanogenerator, circuit model, power management, operator theory, robust right coprime factorization

## Abstract

In this paper, an operator-based voltage control method for TENGs is investigated, achieving output voltage tracking without compensators and uncertainty suppression using robust right coprime factorization. Initially, a comprehensive simulation-capable circuit model for TENGs is developed, integrating their open-circuit voltage and variable capacitance characteristics. This model is implemented to simulate the behavior of TENGs with a rectifier bridge and capacitive load. To address the high-voltage, low-current pulsating nature of TENG outputs, a storage capacitor switching model is designed to effectively transfer the pulsating energy. This switching model is directly connected to a buck converter and operates under a unified control strategy. A complete TENG power management system was established based on this model, incorporating an operator theory-based control strategy. This strategy ensures steady output voltage under varying load conditions without using compensators, thereby reducing disturbances. Simulation results validate the feasibility of the proposed TENG system and the efficacy of the control strategy, providing a robust framework for optimizing TENG energy harvesting and management systems with significant potential for practical applications.

## 1. Introduction

Triboelectric nanogenerators (TENGs) have emerged as a transformative technology in the field of energy harvesting [1], capitalizing on the triboelectric effect to convert mechanical energy into electrical energy. The fundamental operating principle of TENGs involves the generation of electric charges through the contact and subsequent separation of two dissimilar materials. This process, known as contact electrification, leads to the formation of a potential difference between the materials, thereby inducing an electric current when connected to an external circuit. The area power density produced by a TENG has reached as high as 500 W m^−2^, volume power density 15 MW m^−3^, and instantaneous conversion efficiency approximately 70% [2]. TENGs have garnered significant attention due to their high efficiency, cost-effectiveness, and adaptability, making them suitable for a wide range of applications [3,4].

TENGs are classified based on their operational modes and output types, influencing their efficiency and application. The four main modes [5,6,7,8,9,10] are contact–separation, which generates alternating current (AC) through the periodic interaction of two materials; sliding, which provides a more consistent electrical output through continuous sliding motion; single-electrode, which produces electricity through relative movement between a single electrode and a grounded object; and freestanding triboelectric layer, where a mobile layer interacts with a stationary counterpart for optimized energy conversion. Additionally, TENGs are categorized as alternating current (AC) or direct current (DC) types [11,12,13,14]. AC TENGs generate high-voltage, low-current alternating current, suitable for environments with periodic movements but requiring rectification for DC use. DC TENGs produce stable, continuous direct current, ideal for powering low-power electronics without additional rectification. The choice between AC and DC TENGs depends on the application, with ongoing research aimed at improving the efficiency and versatility of both types.

Unlike conventional energy harvesting methods such as piezoelectric, electromagnetic, and thermoelectric generators, TENGs generate electrical charges through the contact and separation of different dielectric materials. This unique mechanism enables TENGs to capture energy from a variety of mechanical sources, including vibrations, human movements, and environmental flows like wind and water. Their high-voltage, low-current output, combined with lightweight and flexible designs, makes TENGs particularly well suited for powering low-energy devices in applications like wearable electronics and remote sensing. Additionally, TENGs’ adaptability to various configurations, such as vertical contact–separation and single-electrode modes, enhances their applicability in scenarios where other energy harvesting technologies might be less effective. In contrast, traditional systems such as piezoelectric generators [15,16], while efficient in converting mechanical stress into electrical energy, are often made from brittle, costly materials, limiting their use in flexible or portable applications. Electromagnetic generators [17], though powerful for large-scale energy conversion, require significant mechanical input and are less suited for compact or lightweight designs. Thermoelectric generators, which rely on heat gradients for power, suffer from low conversion efficiency and require substantial temperature differences. TENGs stand out for their cost-effectiveness, environmental friendliness, and versatility across various scales of energy harvesting, positioning them to potentially complement or surpass traditional methods as research and development continue to advance in this field.

Recent advancements in triboelectric nanogenerators (TENGs) have underscored significant progress in power management [18,19,20,21], focusing on enhanced energy conversion, storage, and regulation systems. Advances in power conversion technology are noteworthy, as sophisticated circuits have been developed to transform the high-voltage alternating current (AC) output from TENGs into stable direct current (DC) power, making it suitable for electronic applications. This development addresses the challenge of integrating TENG outputs with conventional DC-powered devices.

The integration of TENGs with energy storage systems [22,23,24,25], such as supercapacitors and batteries, has also been a focal point of recent research. These advancements have tackled the intermittent nature of TENG outputs by providing a stable and reliable power supply. Combining supercapacitors with batteries has been shown to manage variable output and enhance system reliability. Furthermore, the scalability of TENG technology has been explored, with studies on large-scale fabrication and mass production paving the way for broader commercial and industrial applications. In parallel, significant progress has been made in the development of simulation models, which are crucial for optimizing TENG performance. Detailed modeling of the triboelectric effect in Refs. [26,27] has facilitated better design optimization of TENG systems.

The primary target of this paper is to develop and validate a comprehensive energy management strategy for TENG-based systems, focusing on the integration of PMUs and DC-DC converters. This paper introduces an operator-based voltage control method for TENGs, leveraging theoretical advancements in control theory to address these challenges.

Among various nonlinear control and design techniques including feedback control (FC), linear matrix inequality (LMI) methods, sliding mode control (SMC), and adaptive control (AC), the operator-based robust right coprime factorization method [28,29,30,31,32] stands out for its effectiveness in managing the control and design of nonlinear systems. Operator theory, a sophisticated mathematical framework, is essential for analyzing and controlling nonlinear systems, extending traditional linear algebra methods to handle complex, nonlinear dynamics through the use of operators. This method offers notable advantages, such as eliminating the need for the measurability of all system states—a requirement in many traditional methods. It employs an operator framework within an extended linear space associated with Banach spaces, leveraging the robust tools available in Banach spaces while mitigating the challenges posed by unstable points at infinity. Furthermore, the method ensures bounded input–bounded output (BIBO) stability through adherence to the Bezout identity, making it a compelling choice for addressing complex control engineering problems where full state measurement is often impractical.

This approach is particularly advantageous for managing the intricate interactions between mechanical motion and electrical parameters in TENG-based energy management systems. Specifically, the right coprime factorization (RCF) method within operator theory offers substantial benefits by addressing the nonlinear and time-varying characteristics of TENG outputs [33]. It facilitates the development of robust control strategies that maintain system stability and performance across various operating conditions. The RCF method excels in handling system disturbances and uncertainties, such as fluctuations in mechanical inputs, variations in TENG material properties, and changes in the load connected to the power management unit (PMU). By designing control strategies that are resilient to these disturbances, the RCF method ensures effective operation despite significant variability. Moreover, the inherent flexibility of operator theory allows it to adapt to different TENG and PMU configurations, making it a versatile and powerful tool for optimizing and designing energy harvesting systems across a wide range of applications.

The proposed method employs operator-based right coprime factorization (RCF) to control TENG outputs, providing a novel approach to addressing the nonlinear characteristics of these devices. This strategy focuses on tackling key challenges such as voltage fluctuations, variations in circuit parameters (*L*, *R*, *C*), and ensuring system stability. Initially, a power management system model based on discontinuous conduction mode (DCM) is constructed and subjected to RCF using operator theory. By leveraging Bézout’s identity, a simplified tracking system is designed for precise output voltage tracking. Furthermore, an uncertainty model based on P+ΔP is developed, with an uncertainty suppression strategy from [32] applied to effectively mitigate input voltage disturbances and variations in circuit parameters, thereby ensuring stable output voltage.

While this method shows promise in theoretical simulations, the transition from simulation to practical implementation remains a critical concern. To address these issues, future research will focus on bridging the gap between theoretical models and practical applications. This includes conducting empirical studies to validate the proposed control strategies and designing better compensators to improve system robustness.

The structure of the paper is organized as follows: Section 2 provides a detailed analysis of TENG models, exploring the relationship between theoretical analysis and simulation validation. This section lays the groundwork for understanding the dynamic behavior of TENGs and sets the stage for the subsequent design of PMUs. Section 3 focuses on the design of PMUs, proposing a novel storage capacitor model and validating its performance through both simulation and experimental testing. This section also discusses the integration of the PMU with DC-DC converters, highlighting the challenges and opportunities associated with this integration. Section 4 presents the development of a PMU+DC-DC converter energy collection model, using operator theory and the RCF method to analyze and ensure system stability. This section addresses the issue of voltage fluctuations caused by storage switching and variations in circuit components (*L*, *R*, *C*), and proposes control strategies for output voltage regulation. Section 5 provides simulation verification of the proposed control strategies.

## 2. TENG Modeling

### 2.1. TENG Model and Analysis of Its Circuit Equation

**Figure 1:**
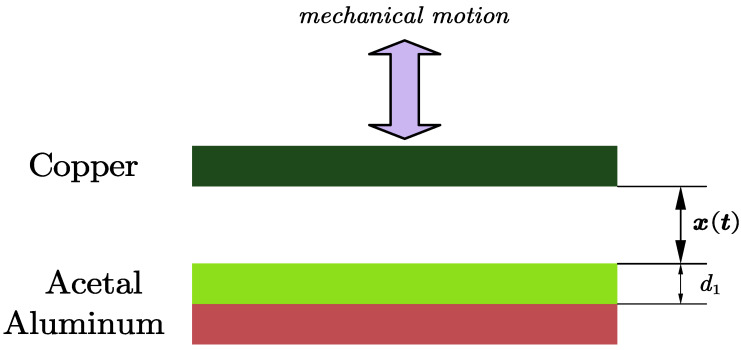
Triboelectric nanogenerator model.

The vertical contact–separation model of a triboelectric nanogenerator (TENG) is primarily investigated, as illustrated in Figure 1. In this model, the upper electrode is copper, while the lower electrode is aluminum, with a layer of acetal dielectric material covering the aluminum. The relevant parameters of the model in Figure 1 are listed in Table 1 and x(t) represents the mechanical motion distance, which varies with time. According to [34], the dielectric material chosen for the study is acetal, with a parameter value that can reach 140 uCm^−2^, which allows the TENG to generate a significant amount of charge during mechanical motion.

**Figure 2:**
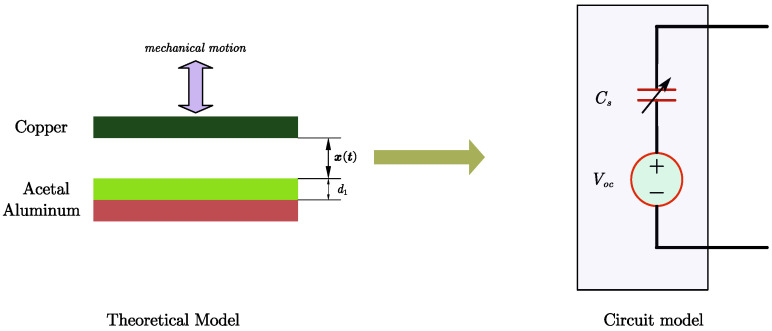
Theoretical and circuit model for vertical contact mode TENG.

Ref. [35] prompts the equivalent circuit model formula with V-Q-X (which means the relationship between voltage, quantity of charge, and separation distance) using an open-circuit voltage (expressed as $V_{oc}$) in series with a variable capacitor (expressed as $C_{s}$), as shown in Figure 2, using Equations (1) and (2). Now, with the highlighting of mechanical motion, the V-Q-X relationship of TENG can be derived as follows (3). From (3), it can be found that the open-circuit voltage, the short-circuit current, and the variable capacitor are closely related to the frequency, rate of change, and amplitude of the externally applied vibration waveform.

(1) $$\begin{array}{c} V_{oc}=\frac{\sigma x(t)}{\epsilon_{0}} \end{array}$$

(2) $$\begin{array}{c} C_{s}=\frac{S\epsilon_{0}}{d_{0}+x\left(t\right)} \end{array}$$

where x(t) represents the separation distance, which changes with time.

(3) $$\begin{array}{cc} \; & V_{oc}\left(t\right)-\frac{Q(t)}{C_{s}}=0\\ \; & V_{oc}\left(t\right)=\frac{\int i(t)}{C_{s}}\\ \; & \frac{\sigma x(t)}{\epsilon_{0}}=\frac{\int i(t)}{\frac{S\epsilon_{0}}{d_{0}+x\left(t\right)}}\\ \; & i\left(t\right)=\frac{d_{0}x^{\prime }\left(t\right)}{{\left(d_{0}+x\left(t\right)\right)}^{2}}=\frac{d_{0}v\left(t\right)}{{\left(d_{0}+x\left(t\right)\right)}^{2}} \end{array}$$

where Q(t) is the charge of transporting in the closed short circuit, and v(t) is the velocity of the mechanical motion.

**Figure 3:**
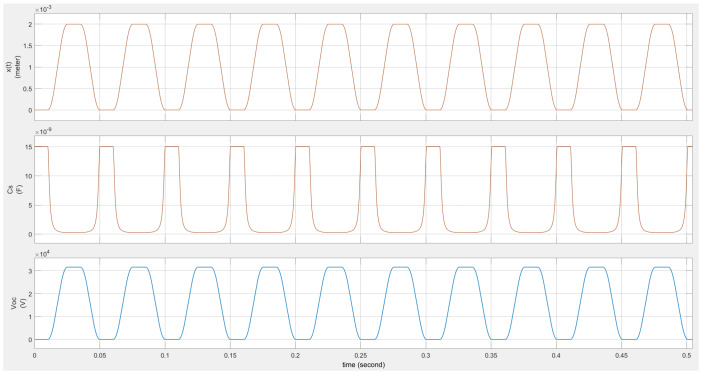
TENG’s $V_{oc}$ and $C_{s}$ values changing with the mechanical motion.

Referring to the external mechanical motion parameters in Table 1 (maximum motion distance of 2 mm, average motion speed of 0.133m/s) and considering that TENGs typically operate in a low-frequency vibration mode, as noted in [36], with frequencies ranging from a few Hz to several tens of Hz, we take the mechanical motion waveform in Figure 3 as an example. This waveform has a frequency of 20Hz and an amplitude representing the maximum distance of mechanical vibration (or the TENG’s upper electrode motion) of 2mm. At the contact point and the highest separation point, the motion is sustained for a short time. The absolute value of the simulated rising and falling speed of the vibration is $\frac{2}{15}\;$m/s, approximately 0.133m/s, which is consistent with the parameters in Table 1.

According to (1) and (2), the $V_{oc}$ and $C_{s}$ waveforms in Figure 3 can be derived to calculate the TENG’s maximum open-circuit voltage and maximum equivalent capacitance, as shown in (4). The results indicate that the TENG has an open-circuit voltage in the KV range (although the power energy is minimal) and an equivalent capacitance in the nF range. These characteristics significantly impact the TENG’s energy output efficiency.

(4) $$\begin{array}{cc} V_{oc(max)} & =\frac{140\times 10^{-6}\times 2\times 10^{-3}}{8.85\times 10^{-12}}=3.16\times 10^{3}\;V\\ C_{s(max)} & =\frac{0.0625\times 8.85\times 10^{-12}}{36.76\times 10^{-6}}=15\;\mathrm{nF} \end{array}$$

**Figure 4:**
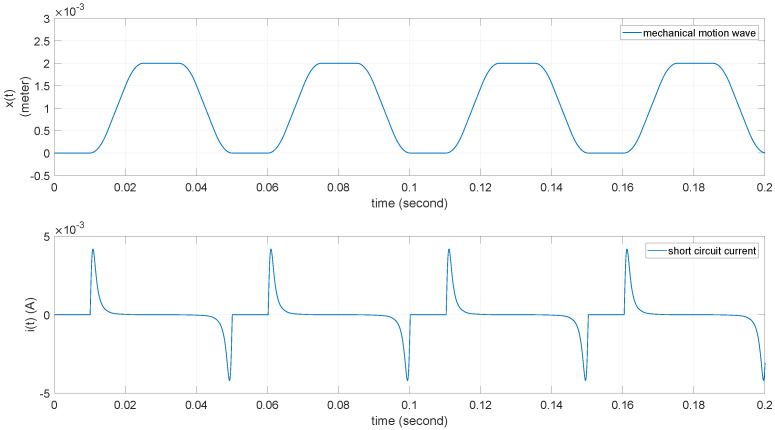
TENG’s short current waveform with mechanical motion.

In the case of an external short circuit on the TENG, the output current can be obtained using (3). Figure 4 shows the short-circuit current waveform of the TENG under the same external mechanical vibration condition.

### 2.2. TENG Simulation Model and Analysis of Its Circuit Equation

The TENG external load can be categorized into resistive load and capacitive load. Ref. [36] provides a detailed analysis of the resistive load case and presents the solutions for voltage and current (as shown in (5) and (6)). However, these formulas are difficult to apply in subsequent analytical solutions, leading to the need for a TENG circuit simulation model. Additionally, the variable capacitor in the TENG’s circuit equivalent model is an inverse function of mechanical motion, making it challenging to describe linearly. Thus, how to design a simulation model for a TENG is one of the research hotspots in the TENG field.

(5) $$\begin{array}{c} V_{oc}\left(t\right)-\frac{Q(t)}{C_{s}\left(t\right)}=V_{out}\left(t\right) \end{array}$$

(6) $$\begin{array}{c} \left(V_{oc}\left(t\right)-V_{out}\left(t\right)\right)C_{s}\left(t\right)=Q\left(t\right)=\int i\left(t\right)\\ i\left(t\right)={\left(\left(V_{oc}\left(t\right)-V_{out}\left(t\right)\right)C_{s}\left(t\right)\right)}^{\prime } \end{array}$$

Ref. [36] presents a $V_{oc}$-$C_{s}$ circuit model study based on the V-Q-X relationship, while [27] provides a simulation circuit model for reconstructing $V_{oc}$-$C_{s}$. It proposes a simulation model design approach through a control flow diagram, but this model requires an additional excitation voltage source, increasing the complexity of the simulation. Based on the control flow design approach from [27] and the $V_{oc}$-$C_{s}$ circuit model from [36], this paper constructs a simulation model that can be used for TENG connections with external circuits such as capacitors, resistors, and rectifier circuits.

**Figure 5:**
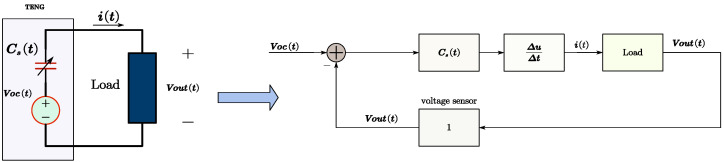
Theoretical TENG model and the simulation model (type 1).

With the V-Q-X relationship of a TENG and the $V_{oc}$ and $C_{s}$ equation, by the KVL and KCL laws, new TENG models can be obtained by setting the load as a black box with consideration of only its $V_{out}$ and i(t) flowing in the closed circuit loop shown in Figure 5, in which $\frac{\Delta u}{\Delta t}$ represents the differential model used to derive i(t). So (5) is derived further to (6).

**Figure 6:**
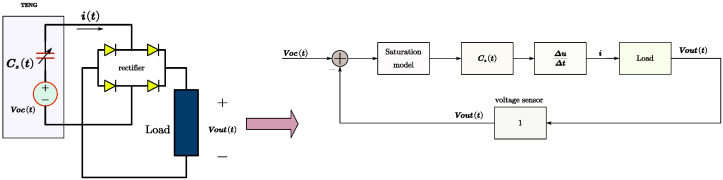
Theoretical TENG model and the simulation model (type 2).

Note that in the expression of (6) and Figure 5, $V_{oc}\left(t\right)$ and $C_{s}\left(t\right)$ are driven and changed by external mechanical motion, $V_{out}\left(t\right)$ is obtained through feedback from a detection sensor, and i(t) is used as a current source to drive the load. It is important to pay attention to the sign of $V_{oc}-V_{out}$, especially when the load includes a rectifier circuit, as shown in Figure 6 with four rectifier diodes. In the case of using rectifiers in a circuit, the unidirectional conductivity of the rectifier circuit and the monotonic increase of the load voltage (particularly under capacitive load conditions) need to be considered. Therefore, (6) should be modified as (7) and (8). In Figure 6, a saturation model is used to express (7) and (8).

(7) $$\begin{array}{c} i\left(t\right)={\left(f\left(\left(V_{oc}\left(t\right)-V_{out}\left(t\right)\right)\right)C_{s}\left(t\right)\right)}^{\prime } \end{array}$$

(8) $$f\left(V_{oc}\left(t\right)-V_{out}\left(t\right)\right)=\left\{\begin{array}{cc} V_{oc}\left(t\right)-V_{out}\left(t\right),V_{oc}\left(t\right)-V_{out}\left(t\right)>0 & \\ 0,V_{oc}\left(t\right)-V_{out}\left(t\right)\leq 0 \end{array}\right.$$

## 3. Proposed TENG’s System and Control

### 3.1. TENG’s System

#### 3.1.1. TENG’s PMU with Storage Capacitor Array

The application of a PMU (power management unit) in a TENG system can filter the pulsed voltage and current outputs of the TENG, providing a stable power input for subsequent converter design. Refs. [15,21,22,23,24] have conducted studies on PMUs. Ref. [15] explains the function of the PMU and designs a self-powered PMU scheme based on a thyristor and Zener diode. When the voltage of the capacitor connected to the TENG output exceeds a set threshold, the capacitor’s charge can flow into the backend DC-DC converter circuit through this PMU. This design requires a relatively high operating voltage, with capacitor voltages mentioned in [15] being above 300 V. This scheme is not suitable for low-voltage operating conditions. Ref. [22] proposes the MR-SCE strategy, which maximizes power transfer efficiency based on a switched capacitor array. This PMU scheme can achieve high transfer efficiency but also introduces complexity in controlling the switched capacitor array. Specifically, it requires the acquisition of the TENG’s pulsed voltage waveform to accurately capture the frequency and waveform characteristics of external mechanical vibrations, which is challenging.

**Figure 7:**
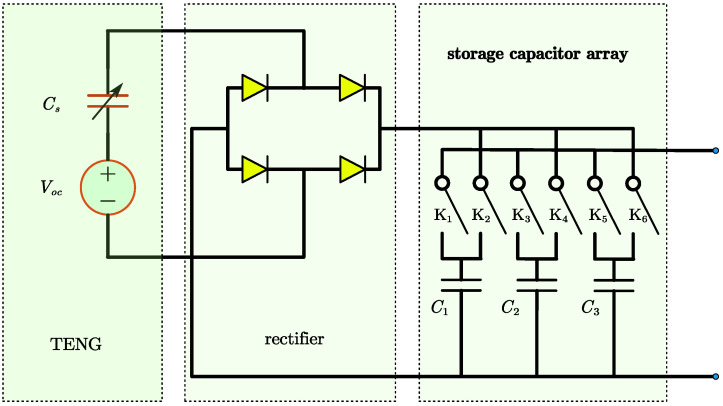
TENG’s PMU design using storage capacitor array.

**Figure 8:**
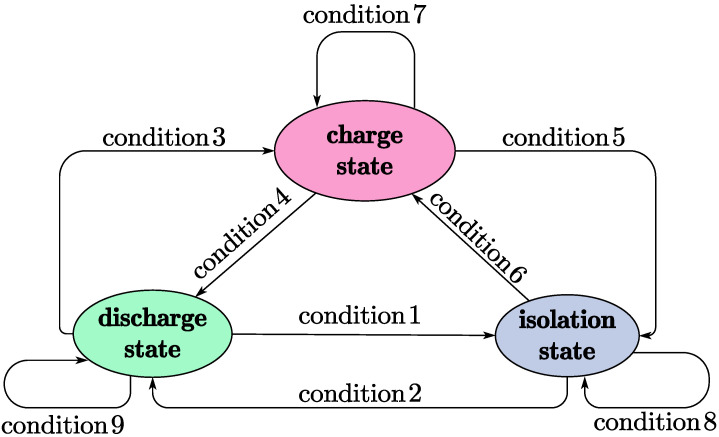
TENG’s storage capacitor array state machine diagram.

Based on [22], this paper proposes a capacitor array dumping scheme, as shown in Figure 7. The storage capacitor array includes three capacitors (expressed as $C_{1}$, $C_{2}$, and $C_{3}$) used to store power energy from the TENG and six switches (expressed as $K_{1}$ to $K_{6}$). The proposed PMU operates in such a way that one capacitor in the array is in a charging state, one capacitor is in a discharging state (serving as the input power source for the backend converter), and one capacitor is in an isolated state. The state transition model of the three capacitors is illustrated in Figure 8 and Table 2. Figure 8 lists the operating states of each capacitor in the capacitor array and the conditions for state transitions. There are three operating states, charging, discharging, and isolation, along with nine transition conditions. The specific state descriptions and transition conditions are detailed in Table 2.

It should be noted that although [22,37] emphasize the efficiency requirements for harvesting TENG energy, it can be seen from (9) that the maximum energy harvesting efficiency is achieved when $C=C_{s}\left(max\right)$. Referring to Figure 3 (under the condition given in Table 1), the maximum value of the variable capacitance within the TENG is in the nF range. To achieve the maximum efficiency for a single energy harvesting, the external capacitor would also need to be in the nF range. According to (10), the charging voltage for one cycle would approach half of the TENG’s maximum voltage (tens of kV in this paper), which is unreasonable and impractical for normal applications.

(9) $$\begin{array}{c} E_{C}=\frac{1}{2}C_{1}V^{2}=\frac{1}{2}C_{1}\frac{{\left(C_{s}V_{oc}\right)}^{2}}{{\left(C_{s}+C_{1}\right)}^{2}}=\frac{1}{2}\frac{C_{s}^{2}V_{oc}^{2}}{C_{1}+\frac{C_{s}^{2}}{C_{1}}+2C_{s}} \end{array}$$

(10) $$\begin{array}{c} V_{out}=V_{oc}\frac{C_{s}}{C_{s}+C_{1}} \end{array}$$

where $C_{1}$ represents the storage capacitor, specifically, the charged capacitor.

**Figure 9:**
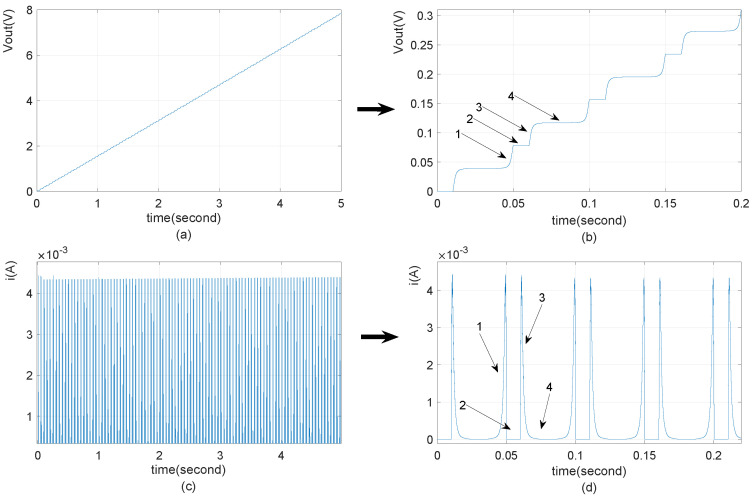
The TENG’s storage capacitor voltage waveform increases when charged (where capacitor value is 220 uF). (**a**) Capacitor voltage increase with time; unit: seconds. (**b**) Capacitor voltage increase with time; unit: 0.05 s. (**c**) Rectified current from TENG with time; unit: seconds. (**d**) Rectified current from TENG with time; unit: 0.05 s.

In the storage capacitor array model, based on the premise that the output storage voltage is much lower than the TENG’s peak output voltage, typically within 100V, the capacitor voltage increases approximately linearly (12), where the voltage rises in steps during each current injection phase. Figure 9 shows the result in which the current frequency is the rectified pulsed current frequency of the TENG, which is twice the TENG’s mechanical vibration frequency. With the rectified current (shown in Figure 9c,d) being charged in the capacitor, the voltage of the charged capacitor increases (shown in Figure 9a,b). Figure 9b,d are time-magnified versions of Figure 9a,c, respectively. From Figure 9b,d, it can be observed that arrows 1 and 3 in Figure 9d represent charging pulse currents, corresponding to the capacitor voltage rise phases in Figure 9b. Arrows 2 and 4 in Figure 9d indicate the absence of pulse current from the TENG, corresponding to the voltage maintenance phases of the capacitor in Figure 9b. In each period of mechanical motion, the circuit equation of $V_{oc}$ and $V_{out}$ can be expressed as (11). Based on (11) and (12) being used to express the voltage of the charging capacitor,

(11) $$\begin{array}{c} V_{oc}\left(t\right)-\frac{Q(t)}{C_{s}}=\frac{Q(t)}{C_{1}}+V_{out}\left(old\right), \end{array}$$

because $V_{out}\ll V_{oc}$, $V_{out}$ can be expressed as

(12) $$\begin{array}{c} V_{out}\left(t\right)=\sum_{i=1}^{n}{\left(\frac{C_{s}}{C_{1}+C_{s}}V_{oc}\left(max\right)\right)}_{i}, \end{array}$$

where $n=\frac{2t}{p}$, *p* is the mechanical motion period, and *t* is the motion time.

Based on the target storage voltage and the known range of external mechanical vibration frequency, the relationship between the specified capacitor load and the storage voltage target value can be calculated (13). This relationship allows for the evaluation of storage capacitor array values, including the target voltage value, charging time, and estimated discharging time, resulting in reasonable design parameters.

(13) $$\begin{array}{c} C_{val}\geq \frac{2t_{request}C_{s}V_{oc}\left(max\right)}{V_{tag}f_{motion}} \end{array}$$

where $t_{request}$ is the maximum charging time which is allowed, $f_{motion}$ is the TENG’s mechanical motion frequency, and $V_{tag}$ is the target storage voltage.

Although the storage capacitor array can provide smooth pulsating voltage and current for the TENG and the backend converter circuit, according to Figure 8 and Table 2, switching of capacitors in the storage capacitor array also introduces new challenges for the backend converter. Specifically, the backend converter needs to handle the impact of the input power transitioning from $V_{c}\left(min\right)$ to $V_{c}\left(max\right)$. This effect is analyzed and addressed as an internal disturbance factor in the subsequent uncertainty suppression design, treated as an operator in the system.

#### 3.1.2. TENG’s DC-DC Converter Structure

**Figure 10:**
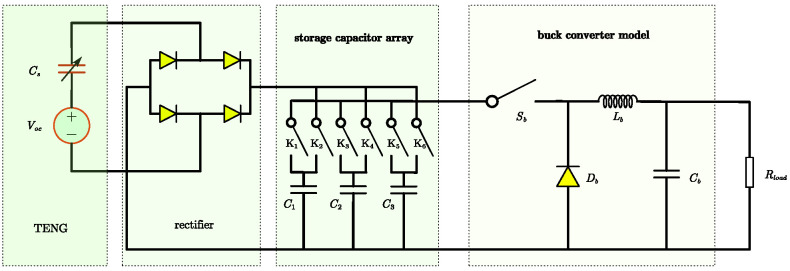
TENG’s system structure with buck converter.

Figure 10 shows a TENG system with a power management unit including a DC-DC buck converter; the storage capacitor array switcher is controlled by a system controller, which is responsible for output voltage, using a specific control strategy. Equation (12) can be re-expressed as (14), and from (14) the controller controls the switching based on the conditions of $V_{c}\left(min\right)$ and $V_{c}\left(max\right)$.

(14) $$\begin{array}{c} V_{out}\left(t\right)=n*U_{c} \end{array}$$

where $U_{c}$ means the voltage increment. This paper assumes that the TENG system has no disturbances or sudden changes in its mechanical vibration process and that the charging process of the storage capacitor is a continuous and stable process as expected. Based on this, the study of the control strategy of the TENG system focuses on the stable tracking of a specified output voltage, including the input power management of the DC-DC converter and counteracting the effects of system parameters’ variations and voltage fluctuations due to input power switching.

(15) $$\begin{array}{c} R_{crit}=\frac{2L_{b}f_{s}}{1-D_{a}} \end{array}$$

where $f_{s}$ is the frequency of PWM, and $L_{b}$ is the inductor used in the buck converter. In Figure 10, a DC-DC buck converter with switch $S_{b}$, inductor $L_{b}$, capacitor $C_{b}$, and flyback diode $D_{s}$ is chosen to achieve a specified lower voltage output ($V_{out}<5\;$V) based on a TENG system. Since the energy output of a TENG is relatively small (in the microwatt range), the load on this system is light ($R_{load}\geq 100\;$KΩ). According to (15)’s criterion, in which $R_{load}$ is obviously greater than $R_{crit}$, the buck converter operates in the discontinuous conduction mode (DCM) region [38,39]. Additionally, the upper limit of the storage capacitor voltage in the system is set to around 40V, so DCM can also meet the requirement for wide voltage range conversion.

**Figure 11:**
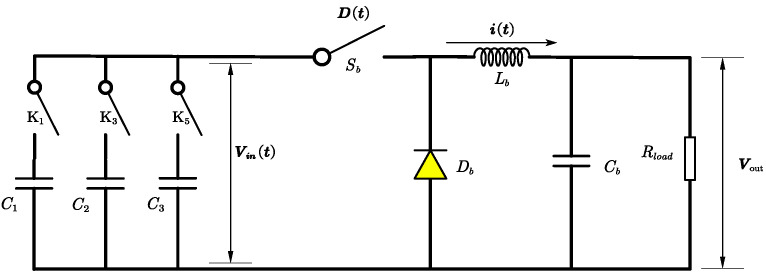
TENG’s buck converter structure.

The circuit of the DC-DC buck converter with capacitor array is depicted in Figure 11. From Figure 11, $V_{in}\left(t\right)$ is the output voltage and used as the input voltage source of the DC-DC buck converter, D(t) is the duty of switch $S_{b}$, i(t) is the current flowing from the inductor $L_{b}$, and $V_{out}$ is the output voltage, which is the controlled objective. Parasitic components are not considered using an ideal switch, inductor, resistor, and capacitor. The regulating operation is managed by the duty ratio D, which controls the switch’s on-and-off state and represents the on-time ratio of the switch within one control period.

**Figure 12:**
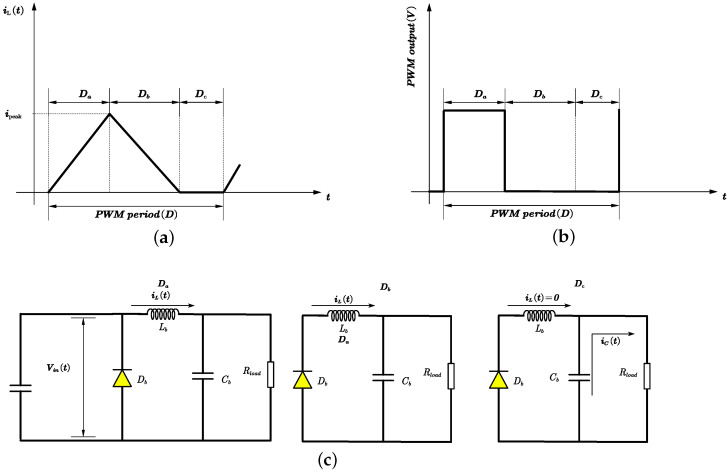
Buck converter DCM in a PWM period. (**a**) The definitions of $D_{a}$, $D_{b}$, and $D_{c}$’s intervals according to the current state. (**b**) $D_{a}$, $D_{b}$, and $D_{c}$’s intervals expressed in a PWM period. (**c**) The circuit states of $D_{a}$, $D_{b}$, and $D_{c}$.

Figure 12 illustrates the DCM mode feature, in which there are three distinct intervals in the inductor current: charging ($D_{a}$), discharging ($D_{b}$), and sleeping ($D_{c}$). The duty ratio of the charging interval is denoted by $D_{a}$, which is also the on time in a PWM period; the discharging interval by $D_{b}$; and the sleeping interval by $D_{c}$; with the relationship $D_{a}+D_{b}+D_{c}=1$. The circuit configuration changes according to these intervals. During the charging interval $D_{a}$, the switch is on and current flows through the inductor, increasing linearly. In the discharging interval $D_{b}$, the switch is off, the diode is on, and the current decreases linearly until it reaches zero. In the sleeping interval $D_{c}$, both the switch and the diode are off, leaving the inductor floating and the output circuit consisting only of the capacitor and load. The inductor current can only be defined during $D_{a}$ and $D_{b}$, making the average inductor current equal to the average current during these intervals, as described in (16) and (17).

(16) $$\begin{array}{c} i_{L}=\frac{i_{peak}}{2}\left(D_{a}+D_{b}\right) \end{array}$$

(17) $$\begin{array}{c} i_{peak}=\frac{v_{in}-v_{out}}{L}D_{a}T_{s} \end{array}$$

where $v_{out}$ denotes the average voltage, which equals the voltage across the capacitor *C* during one PWM period $T_{s}$.

From Figure 12, the combined state equations for the three intervals ($D_{a},D_{b},D_{c}$), by applying the averaging concept, are as shown in (18).

(18) $$\begin{array}{c} \frac{d}{dt}\left[\begin{array}{c} i_{L}\\ v_{out} \end{array}\right]=\left[\begin{array}{cc} 0 & -\frac{D_{a}+D_{b}}{L}\\ \frac{1}{C} & -\frac{1}{CR_{out}} \end{array}\right]\left[\begin{array}{c} i_{L}\\ v_{out} \end{array}\right]+\left[\begin{array}{c} \frac{D_{a}}{L}\\ 0 \end{array}\right]v_{in} \end{array}$$

The relationship between $D_{a}$ and $D_{b}$ is obtained from (16) and (17), as shown in (19). Finally, a state equation that contains only $D_{a}$ is obtained as (20):

(19) $$\begin{array}{c} D_{b}=\frac{2Li_{L}}{D_{a}T_{s}\left(V_{in}-V_{out}\right)}-D_{a} \end{array}$$

(20) $$\left\{\begin{array}{cc} \frac{di_{L}}{dt} & =\frac{D_{a}V_{in}}{L_{b}}-\frac{2i_{L}V_{out}}{D_{a}T_{s}\left(V_{in}-V_{out}\right)}\\ \frac{dV_{out}}{dt} & =\frac{i_{L}}{C_{b}}-\frac{V_{out}}{R_{load}C_{b}} \end{array}\right.$$

(21) $$\begin{array}{c} \frac{d^{2}V_{out}}{dt^{2}}+\frac{dV_{out}}{dt}\left(\frac{1}{R_{load}C_{b}}+\frac{2V_{out}}{D_{a}T_{s}\left(V_{in}-V_{out}\right)}\right)-\frac{V_{out}}{R_{load}C_{b}}=\frac{D_{a}}{L_{b}C_{b}}V_{in} \end{array}$$

Equation (20) is usually called the full-order state-space equation of the buck converter based on the DCM operating mode [40]. In (20), $D_{a}\neq 0$ for the stable equation while, in fact, the DC operating point of the buck converter with a constant duty ratio can be determined by making the right-hand sides of the differential (20) equal to zero and solving the two resulting algebraic equations for $i_{L}$ and $V_{out}$. So, the DC value of $V_{out}$ can be obtained as (22), in which *M* is expressed as (23).

(22) $$V_{out}=V_{in}M$$

(23) $$\begin{array}{c} M=\frac{2}{1+\sqrt{1+\frac{4k}{D_{a}^{2}}}}\\ k=\frac{2L_{b}f_{s}}{R_{load}} \end{array}$$

### 3.2. TENG’s System Control

**Figure 13:**
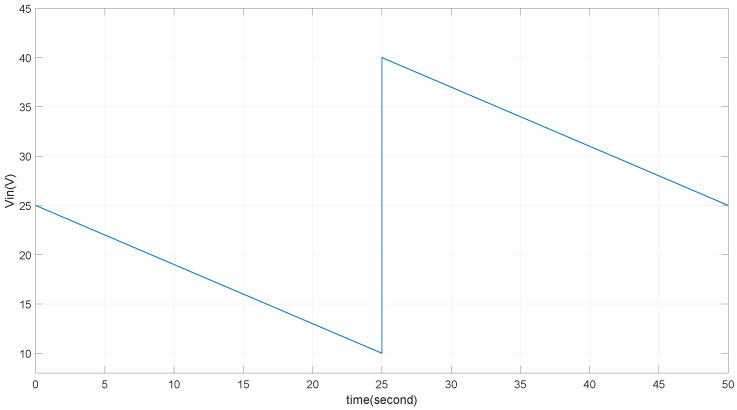
The waveform of the input voltage’s linear decrease and switching.

**Figure 14:**
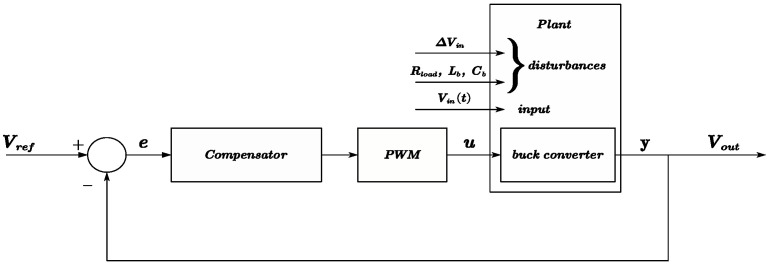
System control flow diagram with uncertainty and disturbance considered.

When the buck converter is operating in DCM mode, the output voltage $V_{\mathrm{out}}$ is a function of the input line voltage $V_{in}\left(t\right)$, the duty cycle $D_{a}\left(t\right)$, and the converter circuit element values such as $L_{b}$, $R_{\mathrm{load}}$, and $C_{b}$, among others. The research objective in the converter circuit is to maintain the output voltage $V_{\mathrm{out}}\left(t\right)=V_{\mathrm{ref}}$ despite disturbances from $V_{in}\left(t\right)$ and variations in the converter circuit element values (referred to as uncertainty). As described before, $V_{in}\left(t\right)$ may appear as the waveform shown in Figure 13, where $V_{in}\left(t\right)$ presents as a saw-tooth waveform, with a maximum voltage of 40V and a minimum voltage set at 10V. During discharging, the voltage changes approximately linearly, with the rate of change depending on the output current of the load. The control diagram is illustrated in Figure 14. The input $V_{in}\left(t\right)$ of an offline power supply from a TENG using a storage capacitor array typically contains periodic variations at a harmonic of several tens of seconds of the switch period of the array.

From Figure 13, it is evident that $V_{in}\left(t\right)$ from the TENG continuously varies, especially considering the small capacitance of its storage capacitors (as limited by (13)). The characteristics of this variation include sustained linear changes and voltage jumps during capacitor switching. In Figure 14, control methods are used to suppress uncertainties in key parameters of the converter circuit, including the individual suppression of uncertainties in the $L_{b}$, $R_{load}$, and $C_{b}$ parameters, as well as the overall uncertainty suppression effect, which help construct a control strategy robust against real circuit component parameter variations. For instance, suppression when the $L_{b}$ parameter fluctuates by 40% around 5 uH, when the $C_{b}$ parameter fluctuates by 50% around 1 uF, and more critically, suppression against variations in the load resistance.

$R_{\mathrm{load}}$ often experiences significant variations and its changes affect the output voltage, forming the load regulation rate in a linear regulation system. The smaller this rate, the stronger the voltage stability capability of the regulation system. This paper explores in depth the suppression effects of variations in $R_{\mathrm{load}}$.

From Figure 14, the converter outputs the voltage $V_{\mathrm{out}}$ based on the input parameters $V_{in}\left(t\right)$ and the duty cycle (here, expressed as *u*), and $V_{\mathrm{out}}$ is fed back and compared to the reference voltage to obtain the error *e* for controlling the compensator adjustment to generate the duty cycle. However, the buck converter circuit in this paper operates in DCM mode. Through the state-space averaging (SSA) method, the state-space expression in (20) and its differential form in (21) reveal that it is a nonlinear system, making it challenging to design control using conventional methods.

In (20), $D_{a}\neq 0$ to ensure that the system equations have a solution. According to the operating principle of a buck converter, $D_{a}=0$ corresponds to no input voltage ($V_{in}=0$), resulting in an output voltage of 0 ($V_{out}=0$). When $D_{a}\equiv 0$, the system is essentially in a non-operational state. Therefore, $D_{a}=0$ needs to be treated as a particular solution in the state-space analysis (SSA) model. The condition for the equation to be held is $D_{a}>0$.

Further analysis reveals that, based on the working principle of the buck converter, the output voltage $V_{out}$ can only be physically meaningful if $V_{out}<V_{in}$. Thus, in the state equations, $V_{in}-V_{out}\leq 0$ is not possible. Given that the system parameters *L*, *R*, and *C* are bounded and not equal to 0 and the input parameter $D_{a}\in \left(0,1\right]$ and the output $V_{out}\in \left[0,\;V_{in}\right)$, so both the input and output both are bounded. Therefore, the state Equation (20) of this system is a bounded-input bounded-output (BIBO) nonlinear system.

#### 3.2.1. Operator-Based TENG System Decomposition

According to the operator theory [28], the plant operator P:U→Y is said to have a right factorization if there exists a linear space *W* and two stable operators D:W→Y and N:W→Y such that *D* is invertible from *U* to *W* and $P=ND^{-1}$ on *U*. This factorization is denoted as (N,D) and *W* is termed a quasi-state space of *P*.

Let (N,D) be a right factorization for P:X→Y:

(24) $$\begin{array}{c} P=ND^{-1},\;N:W\to Y,\;D:W\to U \end{array}$$

Here, *N* and *D* are stable operators from the quasi-state space *W* to the input and output spaces.

*P* is considered a right coprime factorization (RCF) if there exist two stable operators A:Y→U, B:U→U where *B* is invertible. The operators A,N,B,D satisfy the Bezout identity:

(25) $$\begin{array}{c} AN(w)+BD(w)=M(w) \end{array}$$

where M∈U(W,U) and is unimodular. If W=U, typically *M* is replaced by the identity operator *I*.

(26) $$\begin{array}{cc} \; & AN(w)+BD(w)=I(w) \end{array}$$

**Figure 15:**
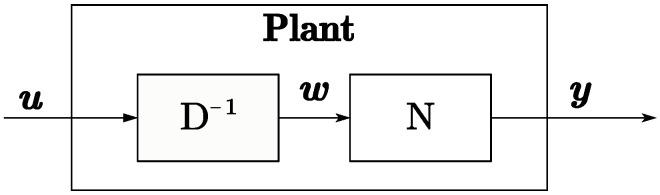
Plant model based on operator theory.

**Figure 16:**
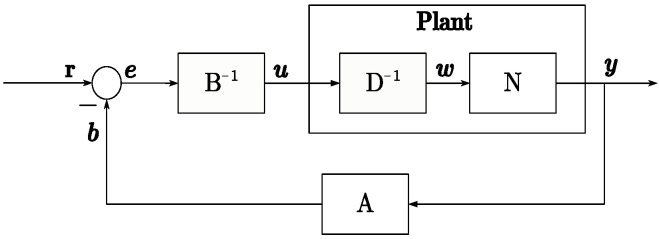
Plant diagram with A,B model.

Using the right coprime factorization method, the plant can be decomposed into stable mapping relationships, providing a method for subsequent tracking design and uncertainty suppression. Figure 15 and Figure 16 depict the system mapping relationships based on RCF.

This paper views the TENG’s buck converter module with discharging storage capacitor as a plant and applies operator theory to decompose it into right coprime factors (RCFs). By analyzing the mapping relationships of the decomposed *D* and *N* matrices, a method for the plant to track the reference input is proposed.

By (26), it is evident that applying the mapping $N^{-1}$ after referencing the input allows for output *y* to track the reference *r*. The key to achieving AN+BD=I lies in its right coprime factorization. Combining (20) and (21), we derive the mappings *N* and $D^{-1}$, as shown in (27) and (28).

(27) $$\begin{array}{c} N\left(w\right)\left(t\right)=\left\{\begin{array}{cc} {\dot{x}}_{n}\left(t\right)=\frac{w}{C_{b}}-\frac{x_{n}\left(t\right)}{R_{load}C_{b}} & \\ y=x_{n} \end{array}\right. \end{array}$$

(28) $$\begin{array}{c} D^{-1}\left(u\right)\left(t\right)=\left\{\begin{array}{cc} {\dot{x}}_{d}\left(t\right)=\frac{w}{C_{b}}-\frac{x_{d}\left(t\right)}{R_{load}C_{b}} & \\ \dot{w}=\frac{V_{in}}{L_{b}}u-\frac{1}{u}\frac{2wx_{d}\left(t\right)}{T_{s}\left(V_{in}-x_{d}\left(t\right)\right)} \end{array}\right. \end{array}$$

In the mapping of *N*, the mapping from *w* to *y* is realized. According to the formula, *w* represents *i*; the state variables $x_{n}\left(t\right)$ are first solved, which also represent the output *y*. In the mapping of $D^{-1}$, the mapping from *u* to *w* is implemented. Here, *u* represents $D_{a}$. The state variables $x_{d}\left(t\right)$ are first solved, and then, *w* is jointly solved using *u* and $x_{d}\left(t\right)$. According to (26), *A* and *B* are decomposed as follows:

- *A* implements the mapping from output *y* to feedback *b*. Here, *b* is constructed as $\frac{1}{10}w$, residing in the same space as *w*, yielding (29).
- $B^{-1}$ implements the mapping from error *e* to input *u*. Given that *b* is structured in the same space as *w*, *e* is also designed to reside in this space, specifically $e=\frac{9}{10}w$, resulting in (30). The construction of *B* is represented as per the formula, facilitating the mapping from *u* to *e*.

(29) $$\begin{array}{c} A\left(y\right)\left(t\right)=\left\{\begin{array}{cc} x_{a}\left(t\right)=r\left(t\right) & \\ b\left(t\right)=\frac{1}{10}\left(C_{b}{\dot{x}}_{a}\left(t\right)+\frac{x_{a}\left(t\right)}{R_{load}}\right) \end{array}\right. \end{array}$$

(30) $$\begin{array}{c} B^{-1}\left(e\right)\left(t\right)=\left\{\begin{array}{cc} x_{b}\left(t\right)=\frac{10}{9}e\left(t\right) & \\ {\dot{\varphi }}_{b}\left(t\right)=\frac{x_{b}}{C_{b}}-\frac{\varphi_{b}}{R_{load}C_{b}} & \\ \alpha =\frac{Vin}{L_{b}},\beta ={\dot{x}}_{b}\left(t\right),\varphi =\frac{2x_{b}\left(t\right)y_{b}\left(t\right)}{T_{s}\left(V_{in}-y_{b}\left(t\right)\right)} & \\ u=\sqrt{{\left(\frac{\beta }{2\alpha }\right)}^{2}+\frac{\varphi }{\alpha }+\frac{\beta }{2\alpha }} \end{array}\right. \end{array}$$

(31) $$\begin{array}{c} B\left(u\right)\left(t\right)=\left\{\begin{array}{cc} {\dot{x}}_{b}\left(t\right)=\frac{V_{in}\left(t\right)}{L_{b}}u-\frac{1}{u}\frac{2x_{b}y_{b}}{T_{s}\left(V_{in}-y_{b}\right)} & \\ {\dot{y}}_{b}=\frac{x_{b}}{C_{b}}-\frac{y_{b}}{R_{load}C_{b}} & \\ e=x_{b}-y_{b} \end{array}\right. \end{array}$$

So, the following result can be obtained:

(32) $$\begin{array}{c} AN(w)+BD(w)=I(w) \end{array}$$

Based on Figure 15, it is evident that (32) realizes an identity mapping from *w* to *e*. However, it is still necessary to consider the response delay factor in the time-domain system. Therefore, the expression of (32) needs to be modified accordingly, as (33).

(33) $$\begin{array}{cc} AN(w)(t)+BD(w)(t)=I(w)(t) & \\ r(t+\Delta t)=I(w)(t) \end{array}$$

#### 3.2.2. Plant Tracking Designed for Reference

Based on the characteristics of the TENG system, DC-DC converters are primarily used to generate a specified output voltage. Common tracking control models for DC-DC converters include PID and SMC, among others. As previously described, the buck converter model in this paper operates in the DCM mode, characterized by a small output current demand and a wide input–output voltage difference. According to (20) and the DC state (23), the output voltage is not only related to the input voltage and duty cycle but also to the system parameters *L*, *R*, and *C*. This significantly increases the complexity of the output voltage tracking control.

**Figure 17:**
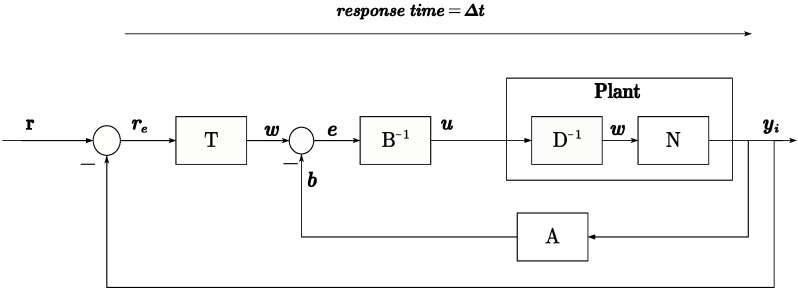
Plant with reference tracking.

The tracking compensator *T* in this paper is depicted in Figure 17. For simplicity, we select $T=N^{-1}$ by decomposing the nonlinear stable system constituted by the buck converter using right coprime factorization (RCF) and operator theory. Therefore, a tracking control system is constructed. So, Figure 16 can be reconstructed as Figure 17 when setting *T* as (34), and the mapping from *r* to *y* can be described as (35).

(34) $$\begin{array}{c} N^{-1}\left(y\right)\left(t\right)=\left\{\begin{array}{cc} x_{m}\left(t\right)=y\left(t\right) & \\ w=C_{b}{\dot{x}}_{m}\left(t\right)+\frac{x_{m}\left(t\right)}{R_{load}} \end{array}\right. \end{array}$$

(35) $$\begin{array}{ccc} \; & y\left(t\right)=N\left(AN+BD\right)N^{-1}\left(r\right)\left(t+\Delta t\right) & \\ \; & y\left(t\right)=NIN^{-1}\left(r\right)\left(t+\Delta t\right) & \\ \; & y\left(t\right)=r\left(t+\Delta t\right)=r\left(t\right)\left(1-e^{-t/T}\right) \end{array}$$

The response time Δt in a time-domain system typically originates from the transient response delay of the system and is usually the coefficient of the output integral term. It can be easily derived as the delay time constant τ of the output y(t) with respect to the reference input r(t) is given by $\tau =R_{\mathrm{load}}C_{b}$. Based on this time constant τ, it is evident that the output response is related to the $R_{\mathrm{load}}$ and $C_{b}$ of the buck converter.

#### 3.2.3. Plant Suppression Designed for Uncertainty

According to the control diagram in Figure 14, the uncertainties of the input $V_{in}$ are shown in Figure 13. During the capacitor discharging period, $V_{in}$ decreases approximately linearly. When $V_{in}$ drops to the minimum voltage $V_{in(\min)}$, it switches to a new capacitor for discharging, causing $V_{in}$ to jump from $V_{in(\min)}$ to $V_{in(\max)}$. The aforementioned $V_{in}$ disturbances ensure that $B^{-1}$ is bounded under the condition that $V_{in(\min)}>V_{out}$.

Therefore, $V_{in}+\Delta V_{in}$ can be re-expressed as $V_{in2}$. With variable substitution, the condition is satisfied. Since *T* and *N* do not include $V_{in}$ terms, tracking control can largely achieve suppression of $V_{in}$.

The primary parameters of a buck converter, namely, *L*, *R*, and *C*, are subject to variations due to nominal discrepancies in electronic component parameters and aging effects from prolonged usage. Furthermore, given that the buck converter discussed herein operates in DCM, the output voltage is intrinsically linked to these component parameters. Consequently, it is imperative to account for the limited uncertainties associated with *L*, *R*, and *C* and to explore appropriate mitigation strategies.

The uncertainties of these three parameters can be expressed as ΔP. Given that *P* has been previously analyzed as a stable nonlinear system, P+ΔP under limited uncertainty is also a nonlinear BIBO system. The limited conditions must satisfy (15), ensuring that R+ΔR does not enter continuous conduction mode (CCM). Additionally, the load cannot be open-circuit, i.e., *R* cannot be infinite, as this would render the output voltage unstable. For simplification, and referring to the tolerance standards of electronic components, ΔL, ΔC, and ΔR are set to ±40% of their nominal values in this paper.

**Figure 18:**
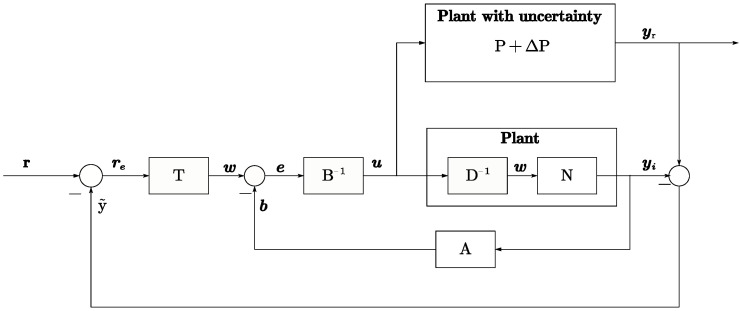
Control diagram of suppression with P+ΔP structure.

This paper proposes a suppression method for uncertainty, which is based on the stability of *P* and the stability conditions of P+ΔP and the thoughts of [32]. By constructing an ideal *P* model and performing feedback control on the output error between *P* and P+ΔP, the suppression of ΔP is achieved. Based on this method, this paper constructs the model shown in Figure 18. Based on the original T+P tracking model, the real P+ΔP is added in parallel with the ideal *P* model to generate the real output $y_{r}\left(t\right)$. The error signal of $y_{i}\left(t\right)-y_{r}\left(t\right)$ is fed back for comparison with *r* to generate the control signal $r_{e}$. As described in Section 3.2.2, $N^{-1}$ is considered as tracking compensator *T* for simplicity. Equation (36) shows that $y_{i}\left(t\right)$ tracks the reference signal r(t) without the effect of ΔP. Equation (37) shows the real $y_{r}\left(t\right)$ output with the time delay factor. Therefore, the suppression of uncertainty from ΔL,ΔR, and ΔC is achieved.

(36) $$\begin{array}{cc} y_{i} & =r-\tilde{y}\\ \tilde{y} & =y_{i}-y_{r}\\ y_{r} & =r \end{array}$$

(37) $$\begin{array}{cc} \; & y_{r}\left(t\right)=r\left(t+\Delta t\right)=r\left(t\right)\left(1-e^{-t/T}\right) \end{array}$$

## 4. Simulation and Verification

### 4.1. Simulation of TENG Model

This paper conducts simulations of a TENG directly connected to capacitive and resistive loads and to RC loads through a rectifier bridge, based on the parameters provided in [26,27]. The results are compared with the corresponding current and voltage outputs.

**Figure 19:**
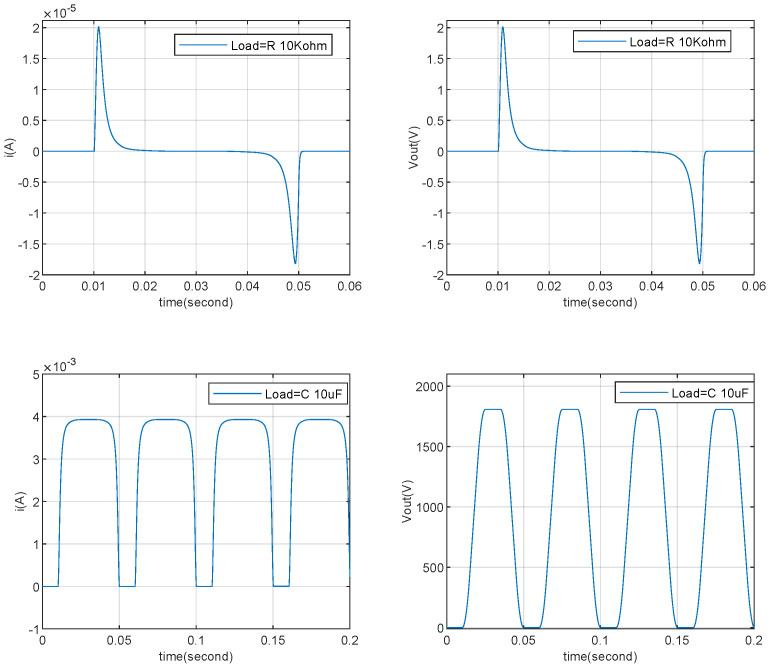
TENG’s capacitor and resistor load simulation verification.

Firstly, the capacitive and resistive load simulations are conducted based on the simulation model in Figure 5. Figure 19 displays the output current and voltage waveforms for a capacitor (C=10 uF) and a resistor (R=100 kΩ). The output current for the resistive load is essentially consistent with the simulated current waveform shown in Figure 2 of [26]. The current output waveform for the capacitive load matches the shape of the current waveform obtained under the capacitive load using the simulation model in Figure 2 of [27]. Since the current and voltage under capacitive load can be calculated using (10) and (38), the numerical results are consistent with the model simulation results.

(38) $$\begin{array}{c} i\left(t\right)={\left(V_{oc}\left(t\right)\frac{C_{load}C_{s}\left(t\right)}{C_{load}+C_{s}\left(t\right)}\right)}^{\prime } \end{array}$$

**Figure 20:**
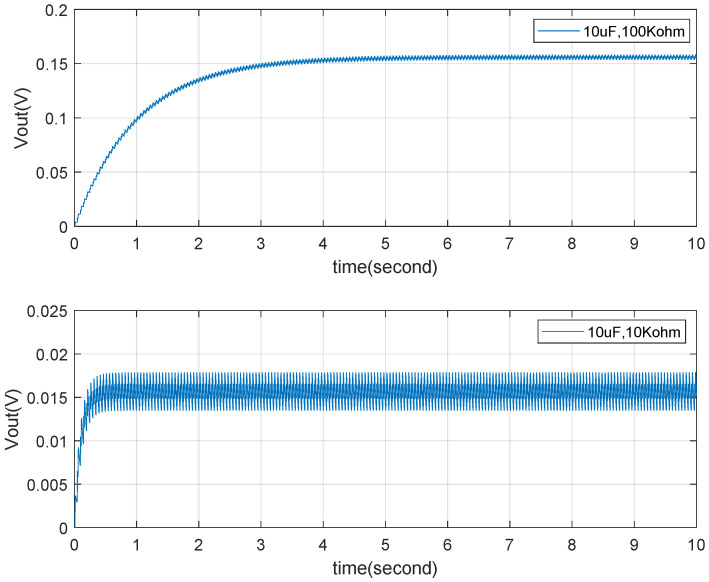
TENG’s RC (C=10uf, R=10kΩ, R=100kΩ) load with rectifier simulation verification.

**Figure 21:**
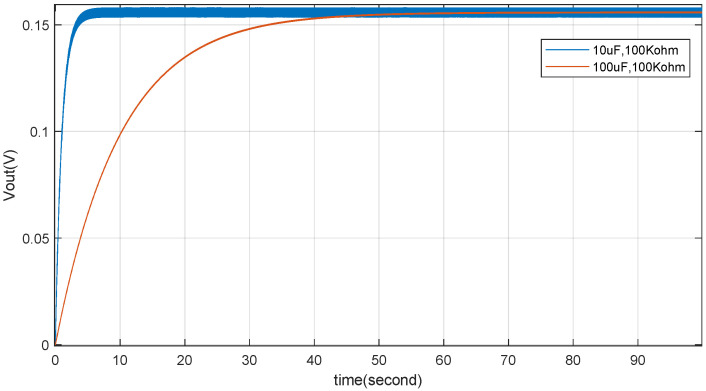
TENG’s RC (C=10uf, C=100uf, R=100kΩ) load with rectifier simulation verification.

Subsequently, simulations of a TENG connected to RC loads through a rectifier bridge are performed based on the model in Figure 6. Figure 20 shows the simulated output voltage waveforms for a capacitor (C=10uF) and resistor (R=10kΩ or R=100kΩ), which are essentially consistent with the voltage output waveforms shown in Figure 3c,d of [26]. Figure 21 presents the voltage output results for capacitors (C=10uF or C=100uF) with a resistor (R=100kΩ), which are essentially consistent with the output results shown in Figure 4d of [26]. The comparison indicates that the simulation model proposed in this paper meets the simulation requirements for a TENG with external RC loads and for a TENG connected to external RC loads through a rectifier bridge.As shown in Figure 21, the amplitude of the output voltage does not fluctuate during the variation of the capacitor $C_{b}$. The primary impact observed is on the tracking response time of the output voltage. Specifically, as the capacitance changes from 10uF to 100uF, the response time increases significantly. This indicates that after the TENGs’ output is rectified the capacitor and load resistor form a charge–discharge circuit. A larger capacitor results in a longer charging time, which translates to a longer response time.

Combining this with Figure 20, it can be seen that the variation in load resistor affects the output voltage fluctuation characteristics when the capacitance remains constant. This fundamentally influences the output current, which in turn causes corresponding changes in the output voltage ripple. A larger load resistor requires less output current, resulting in a smaller voltage ripple. Conversely, a smaller load resistor demands more output current, leading to a larger voltage ripple. Figure 20 reflects this characteristic comprehensively. This provides a foundation for subsequent research on TENG power management.

### 4.2. Simulation of Tracking for Reference

**Figure 22:**
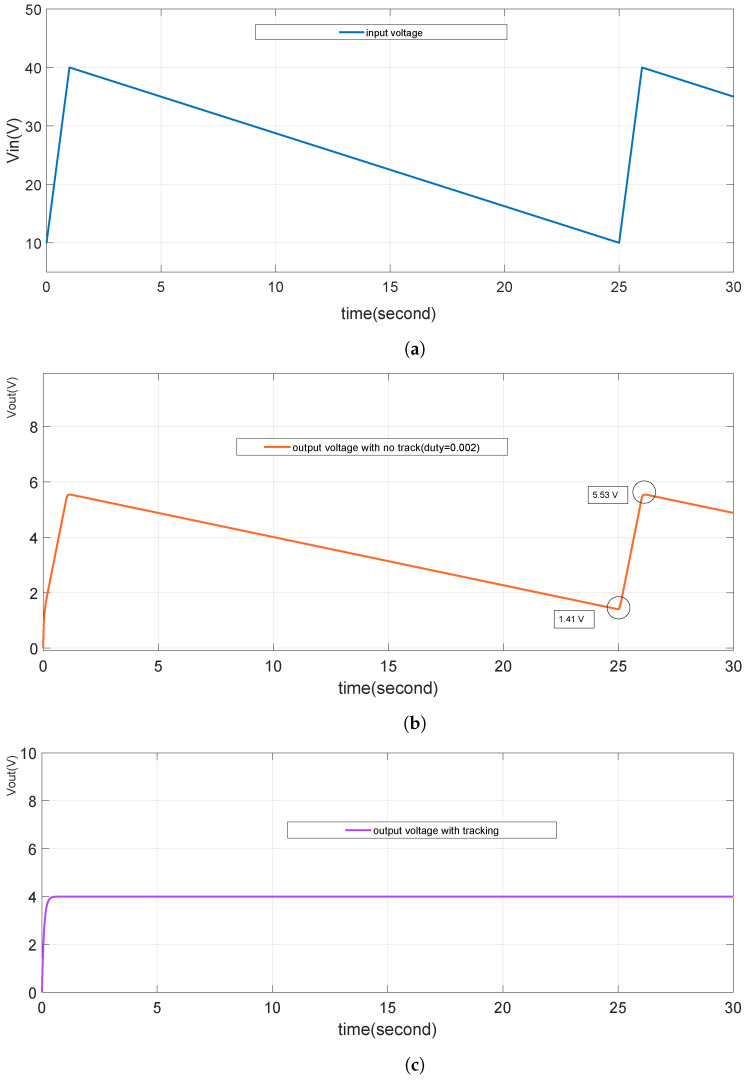
Tracking Vref as 4V. (**a**) is input voltage source with fluctuation between 10 V and 40 V. (**b**) is the output voltage ($V_{out}$) without tracking control. (**c**) is the $V_{out}$ with tracking control.

To evaluate the voltage output tracking performance of the TENG’s power management system, it is considered that the input voltage of the buck converter is sourced from a storage capacitor array. As outlined in Table 3 and discussed earlier, the input voltage fluctuates between 10V and 40V, as depicted in the voltage waveform during the discharge and switching of the storage capacitor in Figure 13. These transitions are realistic and can impact the stable operation of the buck converter. To mitigate these effects, in practice, one solution is to parallel a 1μF capacitor at the input of the buck converter. This capacitor stores energy and helps smooth out voltage jumps during the switching process. As shown in Figure 22, the smoothed input voltage ($V_{\mathrm{in}}$) enables the output to stably track the reference voltage of 4V. Despite periodic variations in the input voltage, particularly during capacitor charging transitions, the system effectively maintains the reference voltage, validating the proposed tracking scheme which is valuable in real circuits when dealing with the storage capacitor’s voltage variation during the discharging and charging states.

**Figure 23:**
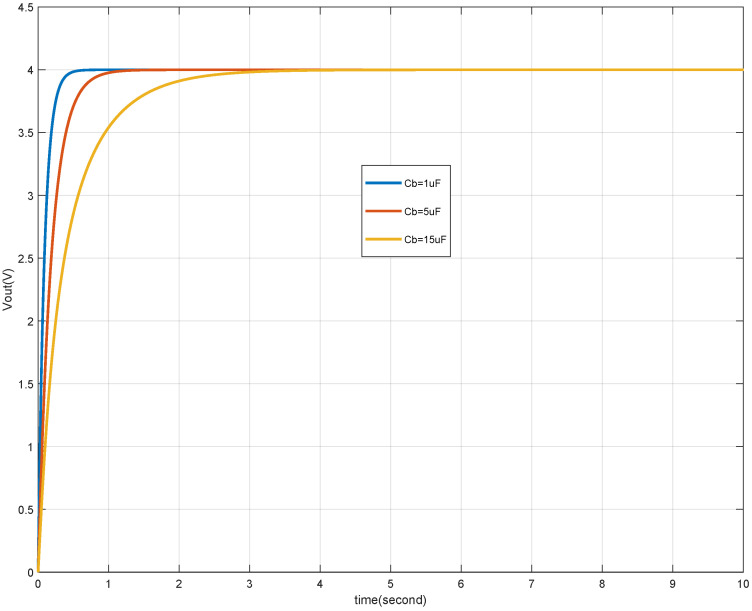
Tracking Vref with different $C_{b}$ values.

The performance of the buck converter’s output tracking is further analyzed by comparing the system response with different capacitance values. Figure 23 highlights that the tracking response time is significantly influenced by the capacitance ($C_{b}$) and the load resistance ($R_{\mathrm{load}}$). With the increase of capacitor $C_{b}$ from 1 uF to 5 uF, the response time of tracking becomes longer, from about 0.5 s to 3 s. Since $R_{\mathrm{load}}$ is a characteristic of the buck converter load, the response time is primarily governed by $C_{b}$, an internal parameter. It is observed that a larger capacitance results in a longer response time, indicating a slower system response. Therefore, careful consideration must be given to the capacitance value to ensure a balance between response time and tracking accuracy. In this study, $C_{b}$ is selected as 1μF, which provides an optimal response time that meets the system’s tracking requirements.

**Figure 24:**
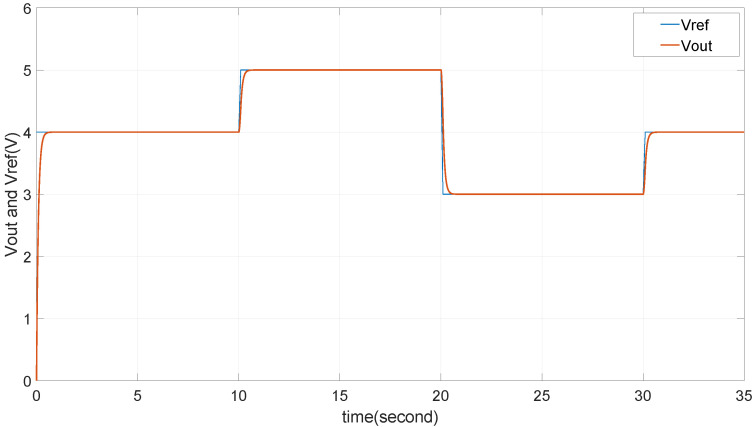
Tracking Vref as 3V, 4V, 5V.

Lastly, the system’s ability to track a fluctuating reference signal is demonstrated in Figure 24, showcasing excellent tracking performance. The output voltage closely follows the reference voltage, even under varying input conditions, which underscores the effectiveness of the power management strategy. This indicates that the proposed design not only smooths the input voltage but also ensures that the buck converter can reliably maintain the desired output voltage. The overall findings confirm that the power management system, with the selected capacitance value, delivers a robust and responsive tracking capability, essential for the stable operation of TENG-based energy systems.

### 4.3. Simulation of Suppression for Uncertainty

**Figure 25:**
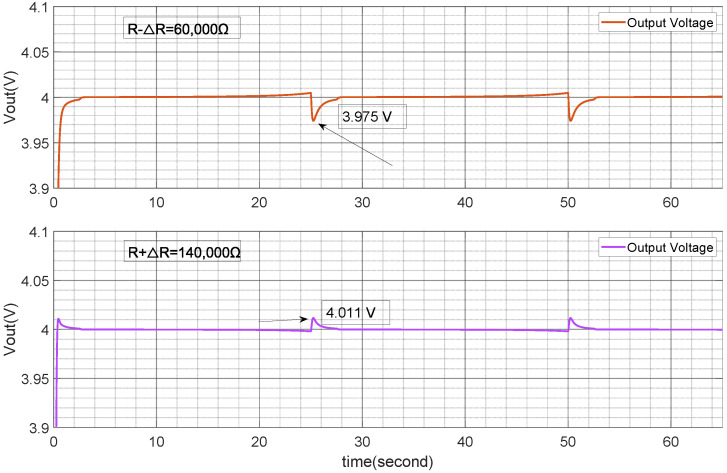
Output voltage with R±ΔR (40,000 Ω).

**Figure 26:**
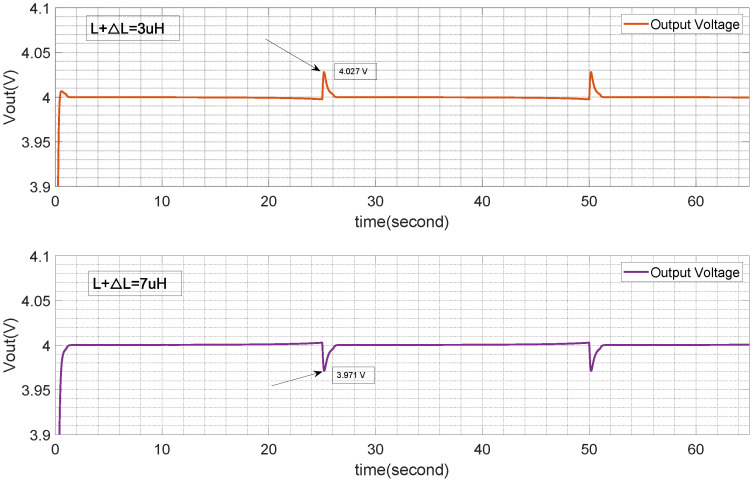
Output voltage with L±ΔL(2uH).

The uncertainty in the TENG system described in this paper is shown in Figure 14, including the input voltage $V_{in}$ and the electronic component parameters *L*, *R*, and *C* of the buck converter. As previously mentioned, the uncertainty in $V_{in}$ can be suppressed through the tracking mechanism, and the tracking effect shown in Figure 22 reflects a good suppression effect on $\Delta V_{in}$. Based on the previous discussion, the uncertainty ranges of ΔL, ΔR, and ΔC are limited to within ±40% for suppression analysis. Figure 25 and Figure 26 demonstrate that based on the input condition $V_{\mathrm{in}}$ (Figure 13), when differences in *L* and *R* arise, output fluctuations around $V_{\mathrm{in}}$ begin to occur in the place switching from $V_{\mathrm{in}(\min)}$ to $V_{\mathrm{in}(\max)}$. These fluctuations are shown in the figure as 0.03V. This indicates that the suppression strategy used can effectively mitigate disturbances originating separately from *L* and *R*, but still experiences the combined effect of these disturbances.

**Figure 27:**
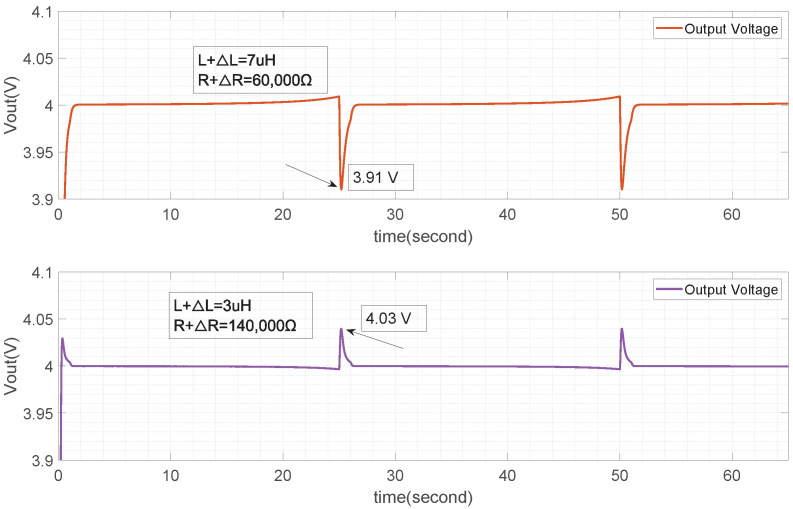
Output voltage with R+ΔR,L+ΔL.

Figure 27 shows the curves after the suppression mechanism are applied under the influence of ΔL and ΔR. Two characteristics can be observed.

Firstly, the impact of ΔC on the system is limited to the response time and within the specified ±40% range; the output tracking effect is unaffected (see Figure 23).

Secondly, ΔL and ΔR have an impact on the system. The comparison of the suppression effects shows that the tracking effect after suppression is better. The fluctuation points are related to the position where the input voltage jumps from 10 V to 40 V. A load adjustment rate analysis is provided to measure the suppression effect under the combined action of ΔL and ΔR. According to Figure 27, the buck converter’s inductor and resistor load value are set as L+ΔL=7μH and R+ΔR= 60,000Ω, the maximum suppressed output ripple can reach 0.09 V. From the viewpoint of load, with the linear adjustment rate definition, the output voltage load adjustment rate can be obtained as $\frac{4-3.91}{4}*100\%=2.25\%$.

Through the simulation of the suppression algorithm for the uncertainty in the input reference tracking and the parameters *L*, *R*, and *C* of the buck converter system, as well as the input voltage $V_{in}$, it can be seen that the proposed controlling algorithm based on operator theory and the right coprime factorization of the TENG power management system can effectively track the reference signal and suppress the input fluctuation of $V_{in}$.

Meanwhile, the uncertainty suppression model built on operator theory can achieve suppression of the uncertainty impacts of the electronic component parameters *L*, *R*, and *C*. The suppression algorithm for the uncertainty impacts of *L*, *R*, and *C* presented in the paper provides strong experimental parameter redundancy for the next step of TENG system prototype verification. This further demonstrates the feasibility of the proposed TENG output voltage control strategy based on operator theory.

## 5. Conclusions

This paper provided an in-depth exploration of TENG systems and their associated power management units. It discussed the operating principles of TENGs, developing a simulation model that accounts for the characteristics of external mechanical vibrations to analyze the open-circuit voltage and short-circuit current. To enhance the performance of the DC-DC converter, the study proposed an energy transfer scheme and an implementation strategy for the storage capacitor array. The operating modes of the buck converter circuit within the TENG system were also examined, leading to the development of a tracking model and an uncertainty suppression strategy using operator theory, based on the state-space averaging method under DCM operation.

The simulation results demonstrated that the proposed tracking and compensator models effectively tracked the reference signal and suppressed uncertainties arising from the primary electrical parameters (*L*, *R*, *C*) of the buck converter. Given that the disturbances in this study stem from the nominal tolerance and aging of component parameters, the simulations hold practical significance.

Due to the low energy output of TENGs, the DC current through the PMU in a DC-DC buck converter is relatively small, causing the buck converter to operate in discontinuous conduction mode (DCM). This makes the control of the buck circuit significantly more complex compared to continuous conduction mode (CCM). For TENG systems that include a power management unit (PMU), the research in this paper on output voltage control based on operator theory and the right coprime factorization (RCF) method is highly relevant. The significance is demonstrated as follows:

1. The buck model in DCM is a nonlinear system model with parameters including inductance, capacitance, duty cycle, input voltage, and output voltage. The multi-parameter nature of the model suggests that the control algorithms will be complex, leading to the need for research on output tracking and disturbance suppression based on this model.
2. This paper applies the RCF method to the buck model, resulting in a simplified tracking compensator. Based on this, a more complex disturbance suppression algorithm is developed using operator theory and validated through simulations, with favorable results. In practical applications, as shown in Figure 18, P+ΔP represents the actual buck circuit, while other parts of the algorithm are based on the buck model (including parameters). By sampling the output voltage and input voltage parameters of P+ΔP, the algorithm can compute the duty signal. This is because the algorithm has already accounted for the effects of device parameter fluctuations (disturbance sources) within the buck circuit, eliminating the need to focus on parameter variations. This design is characterized by its low dependency on the controlled object and high feasibility.
3. Given that TENGs are low-energy-output systems, future research should focus on how to effectively leverage the system’s self-power capability to run the algorithm (based on the controller) while minimizing energy loss and improving the system’s energy utilization efficiency.

The study has limitations, particularly the reliance on simulation models without experimental validation and the simplifications within the current simulation framework. Future research will address these limitations by considering the following directions:

1. Parasitic parameters and model reconstruction: The mathematical model of the buck converter circuit will be reconstructed to include parasitic parameters of key components such as diodes, inductors, capacitors, resistors, and switches. The uncertainty sources will be expanded to encompass these parasitic parameters. Suppression of such uncertainties will be achieved through compensator design based on operator theory.
2. Storage capacitor array model: The design and validation of the storage capacitor array model will be further refined. Testing will be conducted on an experimental platform integrated with the TENG system to ensure that the model accurately reflects real-world performance.
3. Comprehensive system modeling: A detailed mathematical model of the external mechanical vibration source for the TENG will be developed and integrated with the TENG and power system models to create a comprehensive system. Research will focus on the efficient conversion of mechanical vibration energy into electrical energy based on this integrated model.

By addressing these aspects, future work will provide a more robust validation of the proposed strategies and expand the applicability of the findings to real-world TENG systems.

## Figures and Tables

**Table 1:** Parameters of the TENG utilized in this paper.

Parameter	Symbol	Value
Dielectric thickness	$d_{1}$	125 um
Air dielectric constant	$\epsilon_{0}$	8.85 $*\;10^{-12}\;$ F/m
Relative dielectric constant	$\epsilon_{1}$	3.4
Effective dielectric thickness	$d_{0}$	$d_{0}=d_{1}/\epsilon_{1}=36.76\;$um
Width of dielectric	*W*	0.25m
Length of dielectric	*L*	0.25m
Area of dielectric	*S*	S=W∗L=0.0625m^2^
Surface triboelectric charge density	σ	140 uCm^−2^
Maximum separation distance	$x_{max}$	0.002 m
Average velocity of mechanical motion	*v*	0.133 ms^−1^

**Table 2:** TENG’s storage capacitor array state machine definition.

1	State Definition
Discharge state	Storage capacitor is used as power source for DC-DC converter
Charge state	Storage capacitor is charged by TENG
Isolation state	Storage capacitor is not charged and not used as power source
**2**	**Condition Definition**
Condition 1	$V_{c}<V_{c}\left(min\right)$ and TENG is busy
Condition 2	$V_{c}\geq V_{c}\left(max\right)$ and power source is vacancy
Condition 3	$V_{c}<V_{c}\left(min\right)$ and TENG is idle
Condition 4	$V_{c}\geq V_{c}\left(max\right)$ and power source is vacancy
Condition 5	$V_{c}\geq V_{c}\left(max\right)$ and power source is available
Condition 6	$V_{c}<V_{c}\left(min\right)$ and TENG is idle
ine Condition 7	$V_{c}<V_{c}\left(max\right)$
Condition 8	Power source is available and TENG is busy
Condition 9	$V_{c}\geq V_{c}\left(min\right)$

**Table 3:** Parameters of the TENG’s PMU utilized in this paper.

Parameter	Symbol	Value	Unit
Inductor value	$L_{b}$	5	uH
Capacitor value	$C_{b}$	1	uF
Period of PWM	$T_{s}$	1	us
Resistor load	$R_{load}$	100 K	Ω
Voltage of output	$V_{out}$	4	V
Voltage of input	$V_{in}$	See Figure 13	V

## Data Availability

The original contributions presented in the study are included in the article, further inquiries can be directed to the corresponding author.
